# Neurons and Glia Exhibit Distinct Age-Associated MitoTimer-Based Mitochondrial Dynamics in the *Drosophila* Mushroom Body Calyx

**DOI:** 10.17912/micropub.biology.002249

**Published:** 2026-08-18

**Authors:** Allison Walsh, Abigail Wendland, Sofia Uriarte, Caroline Oliynyk, Anitej Akella, Lexxie Padron, Leonard Lorusso, Alice Tsybulski, Nahila Davis, Panteha Sartipi, Casey Spencer

**Affiliations:** 1 College of Science, Department of Biological Sciences, Florida Atlantic, Boca Raton, FL, United States; 2 Harriet L. Wilkes Honors College, Florida Atlantic, Boca Raton, FL, United States

## Abstract

Aging increases the prevalence of diseases with mitochondrial dysfunction, notably Alzheimer’s and Parkinson’s. This study assesses MitoTimer-based mitochondrial dynamics across the lifespan between neurons and glia. MitoTimer was expressed in neurons using nSyb-Gal4 and in glia using Repo-Gal4. MitoTimer red:green fluorescence ratios were quantified within or surrounding the mushroom body calyx of young, middle-aged, and old female *Drosophila melanogaster* . Paraquat-supplemented food increased red:green ratios relative to controls, supporting our use of MitoTimer. Neuronal red:green ratios peaked in middle age, whereas glial ratios were highest in young flies. These findings reveal distinct, age-dependent mitochondrial dynamics in neurons and glia.

**Figure 1. Neurons and Glia Exhibit Distinct Age-Associated MitoTimer-Based Mitochondrial Dynamics in the Drosophila Mushroom Body Calyx:** (A) Mushroom body (MB) ROI selected for the calyx in neurons (nSyb-Gal4) and (B) glia (Repo-Gal4) 20x. Images include green and red MitoTimer, and Brp (far-red, shown here in cyan blue) emission. Scale bar = 20 µm. (C) Schematic illustration of MitoTimer fluorescence from green (unoxidized) to red (oxidized) mitochondria across age groups and cell type. In the MB calyx ROI, neurons display the highest red:green ratio in the middle-aged samples, while glia display the highest red:green ratio in young-aged samples. (D) Paraquat’s effect on mitochondrial redox state illustrated to display the resulting MitoTimer red:green fluorescence ratios. (E) Paraquat-treated and control samples driving UAS-MitoTimer using nSyb-Gal4 (in neurons) and Repo-Gal4 (in glia) shows increased red:green fluorescence ratio under paraquat-induced oxidative damage. Graph displays MitoTimer red:green fluorescence ratio of individual calyces. Student’s t-test was used to report significance, ****p<0.001, ***p<0.005, **p<0.01, *p<0.05. (F) Representative images of nSyb-Gal4/UAS-MitoTimer in young (1 to 5 day), (G) middle (15 to 20 day), and (H) old-aged (45 to 50 day) flies. Images include both green and red MitoTimer emission. Scale bar = 15 µm. (I) Quantification of MitoTimer red:green fluorescence ratio in neurons across ages. Graph displays MitoTimer red:green fluorescence ratio of individual calyces. One-way ANOVA followed by Tukey’s multiple-comparison test (J) Representative images of Repo-Gal4/UAS-MitoTimer in young (1 to 5 day), (K) middle (15 to 20 day), and (L) old-aged (45 to 50 day) flies. Images include both green and red MitoTimer emission. Scale bar = 15 µm. (M) Quantification of MitoTimer red:green fluorescence ratio in glia across ages. Graph displays MitoTimer red:green fluorescence ratio of individual calyces. One-way ANOVA followed by Tukey’s multiple-comparison test (N) Graph displays absolute integrated density (AU) in neurons and (O) glia across age groups. No statistically significant changes were observed in green-MitoTimer integrated density across ages in neurons or glia. In neurons, red MitoTimer integrated density was statistically significant between young and middle age, and middle and old age, while no significance was observed in the young to old age groups. In glia, no significance was observed when comparing any age groups based on red MitoTimer integrated density alone. One-way ANOVA followed by Tukey’s multiple-comparison test. Each data point represents one MB calyx ROI.

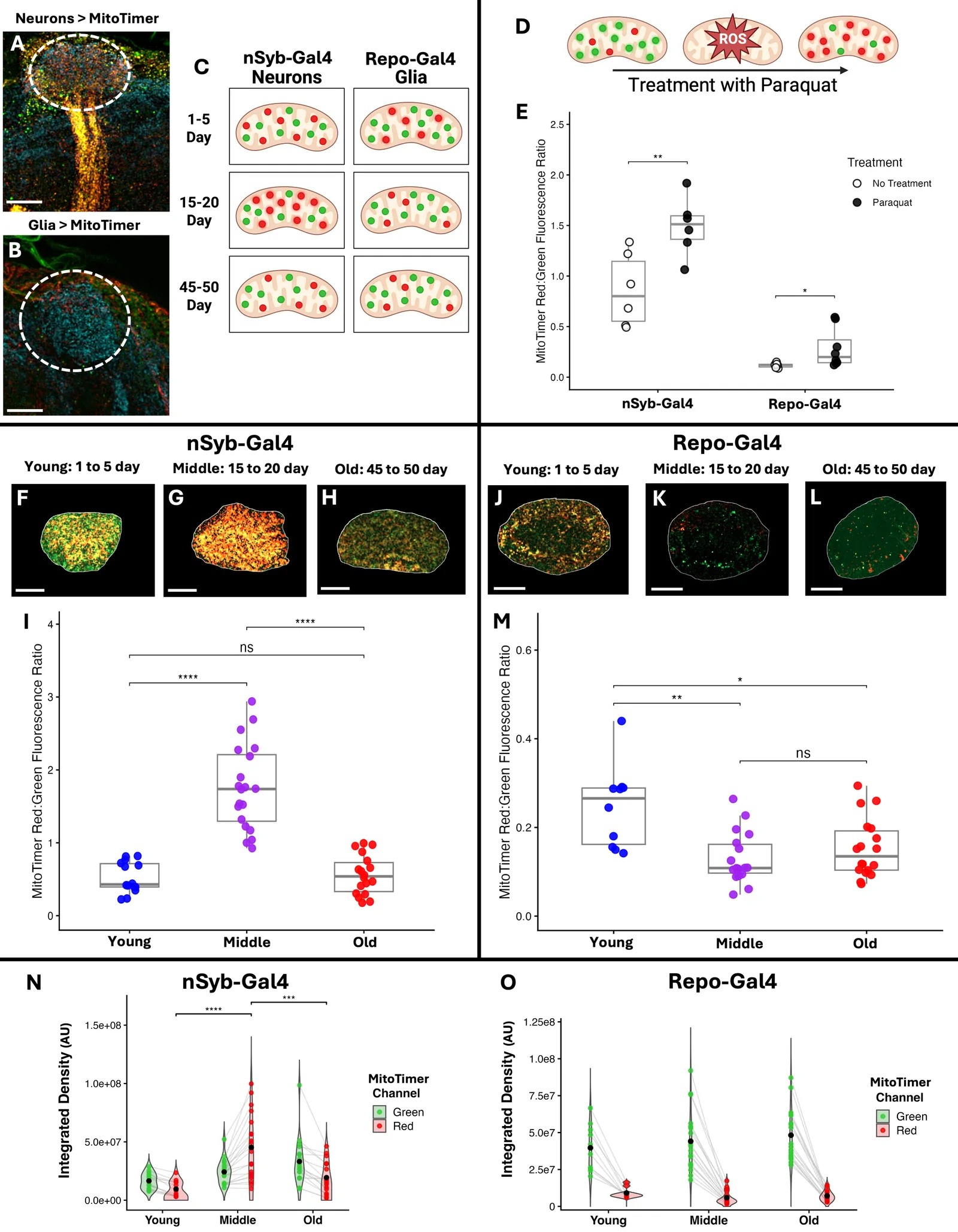



## Description

Here, we leveraged the *Drosophila melanogaster* mushroom body (MB) as a model to characterize mitochondrial dynamics in neurons and supporting glia across the lifespan within a memory-associated brain region. Our comparative analysis shows that mitochondrial dynamics, through MitoTimer red:green fluorescence ratio, differ between neurons and glia across age in the MB calyx of female flies.

The MB is crucial for short- and long-term memory associated with olfactory and visual cues (Quinn et al., 1974; Tully and Quinn, 1985), as well as appetitive and aversive learning (Mao and Davis, 2009; Liu and Davis, 2009; Kremer et al., 2010). MB circuitry includes primarily the Kenyon cells (KCs), intrinsic neurons (cells’ processes contained within a single neuropil), whose cell bodies surround the calyx (Aso et al., 2014; Scheffer et al., 2020; Schlegel et al., 2024; Robinson et al., 2025).

KCs extend dendritic arbors into the MB calyx, a region highly enriched in synaptic connectivity and implicated in experience-dependent structural plasticity (Baltruschat et al., 2021). In addition to the numerous KCs, there are glial cells which aid in axon pruning, metabolic homeostasis, neurotransmitter signaling, neuronal survival, and synaptic plasticity (Puñal et al., 2021; Freeman 2015; Ng et al., 2011). More specifically, surrounding the calyx are cortex glia and ensheathing glia. In addition, astrocyte-like glia (ALGs) project processes throughout the calyx and neighboring neuropil regions. Together, these glial populations establish a complex cellular environment surrounding the calyx.

Aging is associated with impairments in multiple forms of MB-dependent memory, including intermediate- and long-term memory (Tonoki and Davis, 2012; Tonoki and Davis, 2015). Neurons and glia exhibit distinct metabolic and homeostatic demands that may differentially influence mitochondrial redox and turnover dynamics during aging. Because mitochondrial function is tightly coupled to neuronal and glial physiology, age-associated alterations in mitochondrial dynamics may contribute to declining MB circuit function. Recent work further supports a role for neuron-glia metabolic coupling in memory, including studies showing that glial-derived metabolites can support neuronal mitochondrial metabolism during memory formation and that altered neuronal or glial lactate metabolism can affect memory and aging in flies (Frame et al., 2023; Rabah et al., 2023). However, despite established roles for neurons and glia in MB function, comparatively little is known about how mitochondrial dynamics differ between these cell types around the aging calyx ( Figure 1A, B). Mitochondrial dynamics encompass the relative balance between pro-oxidizing species, antioxidant systems, and redox enzymes, together with the biogenesis and turnover of mitochondria within cells (Starkov 2008; Popov 2020; Willems et al., 2015).

To assess the mitochondrial dynamics across aging and cell type in a memory-associated region of the *Drosophila* brain, we expressed the genetically encoded mitochondrial sensor, MitoTimer, using the pan-neuronal nSyb-Gal4 or the pan-glial Repo-Gal4 driver. The ratio of red-to-green MitoTimer fluorescence is an indicator of mitochondrial dynamics ( Figure 1C ). Therefore, we sought to track the abundance of new (green) MitoTimer and oxidized (red) MitoTimer (Hernandez et al., 2013; Laker et al., 2014). Using an antibody against presynaptic active zones (anti-Brp, nc82), we identified the MB lobes and calyx to establish the ROI ( Figure 1A, B). From there, we quantified MitoTimer red:green fluorescence ratios in cells and processes surrounding the MB calyx of female flies. To evaluate mitochondrial dynamics across the lifespan, we examined young (1 to 5 day), middle-aged (15 to 20 day), and old (45 to 50 day) flies in both neurons and glia.

To test whether MitoTimer red:green fluorescence ratio reflects redox state as a component of mitochondrial dynamics in this system, we compared vehicle-control flies with flies raised on paraquat-supplemented media. Paraquat has been shown to induce mitochondrial dysfunction and increase ROS production (Rzezniczak et al., 2011). Standard Bloomington recipe fly food was supplemented with 5 mM paraquat, and 10 to 15 day old flies were maintained on control or paraquat-supplemented food for 24 hours at 25°C. Flies fed a paraquat-supplemented diet showed increased red MitoTimer fluorescence in both neurons and glia ( Figure 1D- E). In nSyb-Gal4/UAS-MitoTimer flies, neuronal MitoTimer expression in the calyx after paraquat supplementation resulted in an increased red:green ratio (paraquat-supplemented: n = 6, mean = 1.49; vehicle control: n = 6, mean = 0.860, p = 0.00785, Student’s t-test) ( Figure 1E ). Similarly, in Repo-Gal4/UAS-MitoTimer flies, glial MitoTimer expression within and around the calyx after paraquat supplementation resulted in an increased red:green ratio (paraquat-supplemented: n = 8, mean = 0.284; vehicle control: n = 6, mean = 0.117, p = 0.0462, Student’s t-test) ( Figure 1E ).

At the outset of this study, we hypothesized that the trajectory of MitoTimer-based mitochondrial dynamics in the aging organism would be associated with a linear increase in red MitoTimer fluorescence and a linear decrease in green MitoTimer fluorescence in both neurons and glia. However, this is not what we observed. We quantified the highest red:green fluorescence ratio in neuronal expression in the calyx of middle-aged (15 to 20 day) flies (Young: n = 14, mean = 0.409; Middle-age: n = 20, mean = 0.985; Old: n = 18, mean = 0.356; Young vs. Middle adjusted p = 6.61e-11 ; Young vs. Old adjusted p = 0.97; Middle vs. Old adjusted p = 1.58e-11; one-way ANOVA followed by Tukey’s multiple-comparison test) ( Figure 1F- I). In contrast, the highest red:green fluorescence ratio in glia was the young (1 to 5 day) flies (Young: n = 10, mean = 0.247; Middle-age: n = 18, mean = 0.130; Old: n = 18, mean = 0.152; Young vs. Middle adjusted p= 0.000319; Young vs. Old adjusted p = 0.0063; Middle vs. Old adjusted p = 0.606, one-way ANOVA followed by Tukey’s multiple-comparison test) ( Figure 1J- M). These findings indicate that aging is not associated with a simple linear increase in mitochondrial redox state or mitochondrial turnover around the MB calyx. Instead, neurons and glia exhibit distinct age-associated mitochondrial dynamics, driven by distinct redox state trajectories and/or the rate of mitochondrial turnover.

Moving forward, clarification of underlying mechanisms could include measures of metabolic activity, metabolic demand, or mitochondrial abundance in each cell type. Future studies could also expand upon this work by directly measuring ROS levels within each cell type and markers of protein/lipid oxidation. Additionally, studies could benefit by expanding the number of age cohorts across the lifespan, such as a shorter time window for young flies and additional older cohorts. Further, the inclusion of both male and female sexes would be useful to determine sex-specific phenotypes that may exist.

Age-related changes in Gal4 driver expression may influence reporter levels and should be considered (Delandre et al., 2025). To address potential age-dependent reporter loss, we quantified the absolute integrated density of green MitoTimer fluorescence separately from the red:green ratio. Green MitoTimer integrated density did not differ significantly across age groups in either neurons ( Figure 1N ) or glia ( Figure 1O ). In contrast, red MitoTimer integrated density showed age-associated differences in neurons, with significant differences between young and middle-aged flies and between middle-aged and old flies, but not between young and old flies (Young: n = 14, mean = 9578269 AU; Middle-age: n = 20, mean = 45301550 AU; Old: n = 18, mean = 19380111 AU; Young vs. Middle adjusted p = 5.87e-6; Young vs. Old adjusted p = 0.325; Middle vs. Old adjusted p = 0.000329, one-way ANOVA followed by Tukey’s multiple-comparison test). Red MitoTimer integrated density did not differ significantly across age groups in glia, indicating that the glial age-associated phenotype was detected primarily in the red:green fluorescence ratio rather than in red fluorescence intensity alone. Overall, this suggests that the observed red:green fluorescence ratio differences are unlikely to be explained solely by age-dependent loss of reporter expression. Thus, while the specific metabolic mechanisms underlying age-associated mitochondrial dynamics remain to be elucidated, our findings support a model in which neuronal and glial mitochondria exhibit distinct MitoTimer-based mitochondrial dynamics throughout the lifespan.

## Methods

Fly Rearing and Genetics

The GAL4/UAS system was employed to drive expression of the MitoTimer reporter in a cell-type-specific manner. Pan-neuronal expression was achieved using nSyb-Gal4 (RRID:BDSC_51635), while pan-glial expression was driven by Repo-Gal4 (RRID:BDSC_7415). These drivers were crossed to UAS-MitoTimer (RRID:BDSC_57323) flies to generate progeny of the genotypes nSyb-Gal4/UAS-MitoTimer and Repo-Gal4/UAS-MitoTimer. Flies were maintained at 25 °C under standard conditions. Crosses were established in a staggered manner to generate synchronized age groups. For the experimental groups, after eclosion, three age cohorts were sampled: young (1 to 5 day), middle-aged (15 to 20 day), and old (45 to 50 day). For the positive control experiments, one age group was sampled: 10 to 15 day old flies. To control for sex-specific differences, only female flies were sampled.

Positive Control Design

To induce oxidative stress as a positive control, flies were fed 5 mM paraquat (methyl viologen hydrate; Acros Organics, CAS: 1910-42-5) - supplemented food for 24 hours prior to dissection. A 100 mM stock solution of paraquat dissolved in Nanopure water was used to supplement the food. 500 μL of 100mM paraquat stock was added to 9.5 mL of melted fly food. After microwaving the fly food, it was pipetted into fresh vials and left to cool briefly before adding 500 μL of 100 mM paraquat (experimental) or 500 μL of water (vehicle control), then mixing with a stir rod. After the supplemented food cooled, flies were briefly anesthetized with CO2 and added to vials. Flies were kept at 25 ºC for 24 hours on supplemented food vials. Following this feeding period, the flies were briefly anesthetized with CO2 and immediately dissected in O’Dowd’s saline. Vehicle control (water-supplemented food) flies maintained under identical conditions without paraquat exposure served as negative controls. Comparisons were made between the treatment and control groups within identical age groups (10 to 15 day) for neuronal and glial populations.

Dissection

Fly brains were dissected in ice-cold O’Dowd’s saline to preserve tissue integrity. Flies were briefly anesthetized with CO2 for dissection. Using a Zeiss Stemi 305 stereomicroscope, the legs, wings, and the proboscis were removed, and flies were pinned dorsal side up in Sylgard-coated dishes using minuten pins. Flies were then covered with O'Dowd's saline, and the air bubble surrounding the head/thorax/abdomen was removed with a syringe. Exposure of the brain was performed using forceps to remove the head cuticle. To ensure consistency among samples, flies were dissected individually and immediately fixed using 4% PFA PBS. Samples were discarded if the brains were damaged during dissection or if the procedure took longer than 3 minutes.

Fixation and Immunohistochemistry

Dissected samples were fixed in 4% paraformaldehyde (PFA) in PBS for 30 minutes at 4 ºC. Samples were washed in PBS and permeabilized in PBS containing 0.5% Triton X-100 for 1.5 hours on a rotator. Tissues were incubated with primary antibody solution containing mouse anti-Brp (1:30; DSHB Cat# nc82, RRID:AB_2314866) diluted in PBS supplemented with 3% bovine serum albumin (BSA) and 0.3% Triton X-100 for 24 hours at 4 °C. Following primary incubation, samples were washed in PBS for 4 hours at room temperature with solution changes every 30 minutes. Secondary antibody incubation was performed using goat anti-mouse Alexa Fluor 647 (1:125; Jackson ImmunoResearch Labs Cat# 115-605-003, RRID:AB_2338902) in PBS for 24 hours at 4 °C. Samples were then washed again in PBS for 4 hours. Following staining, samples were dehydrated through an ethanol series (50%, 70%, 90%, and 100%) for 8 minutes per step. Samples were then cleared and mounted in 100% methyl salicylate on slides and imaged within 24 hours.

Confocal Imaging

Fluorescence imaging was performed using a ZEISS LSM 990 confocal microscope equipped with an Airyscan 2 detector on an AxioObserver Z.1 platform. Images were acquired using a 20×/0.8 NA objective. Laser excitation wavelengths of 488 nm, 568 nm, and 639 nm were used to capture MitoTimer green fluorescence, red fluorescence, and nc82 far-red labeling, respectively. Imaging parameters, including laser power, gain, and exposure time, were kept constant across all samples imaged to ensure comparability. Images were acquired using Airyscan multiplex mode (SR-4Y) with a sampling rate of 2.0. Post-acquisition processing was performed using the Airyscan 3D processing pipeline in ZEN Blue software (v3.13).

Image Analysis and Quantification

Image analysis was conducted using a standardized workflow in ImageJ/FIJI (v1.54p). All Z-stack file names and treatment/ages were blinded to researchers during analysis. Regions of interest (ROIs) corresponding to neuronal or glial populations were identified based on GAL4-driven expression and nc82 labeling to identify each individual (right/left) MB calyx. Mitochondrial dynamics was quantified using the ratio of red to green fluorescence intensity (MitoTimer red:green ratio). The Z-dimension was scanned and cropped to ensure only the ROI was included for each MB calyx region. Integrated density values for red and green channels were extracted for each ROI. Each ROI analyzed represents a single MB calyx. After analysis, samples were unblinded, and ratios were calculated for each sample and grouped by cell type (neurons vs glia), treatment condition (control and paraquat), and age group (young, middle-aged, and old).

Statistical Analysis

All statistical analyses and data visualization were performed in R (v4.6.0). For paraquat experiments, vehicle-control and paraquat-treated groups were compared within each driver using a Student’s t-test. For aging experiments, age groups were compared within each driver using one-way ANOVA followed by Tukey’s multiple-comparison test, and adjusted p-values are reported. Statistical significance was set at p < 0.05. Graphical outputs, including bar plots with error bars and significance annotations, were generated using R packages. All analysis scripts and workflows are publicly available in a GitHub repository to ensure reproducibility.

Github: https://github.com/CLSpencer-Lab/MitoTimer-Calyx
